# Noncanonical Poly(A) Polymerase TENT4 Drives Expression of Subgenomic Hepatitis A Virus RNAs in Infected Cells

**DOI:** 10.3390/v17050665

**Published:** 2025-05-02

**Authors:** You Li, Ankit Gupta, Brian N. Papas, David Aponte-Diaz, Jayden M. Harris, Ichiro Misumi, Jason K. Whitmire, Craig E. Cameron, Marcos Morgan, Stanley M. Lemon

**Affiliations:** 1 Department of Pediatrics, The University of North Carolina at Chapel Hill, Chapel Hill, NC 27599, USA; 2 Reproductive and Developmental Biology Laboratory, National Institute of Environmental Health Sciences, National Institutes of Health, Durham, NC 27709, USA; ankit.gupta2@nih.gov (A.G.); marcos.morgan@nih.gov (M.M.); 3 Integrative Bioinformatics, Biostatistics and Computational Biology Branch, National Institute of Environmental Health Sciences, National Institutes of Health, Durham, NC 27709, USA; brian.papas@nih.gov; 4 Department of Microbiology & Immunology, The University of North Carolina at Chapel Hill, Chapel Hill, NC 27599, USA; dapontediaz@unc.edu (D.A.-D.); jayden.harris@emory.edu (J.M.H.); craig_cameron@med.unc.edu (C.E.C.); 5 Department of Genetics, The University of North Carolina at Chapel Hill, Chapel Hill, NC 27599, USA; imisumi@email.unc.edu (I.M.); jwhitmir@email.unc.edu (J.K.W.); 6 Lineberger Comprehensive Cancer Center, The University of North Carolina at Chapel Hill, Chapel Hill, NC 27599, USA; 7 Department of Medicine, The University of North Carolina at Chapel Hill, Chapel Hill, NC 27599, USA

**Keywords:** nanopore sequencing, hepatovirus, picornavirus, terminal nucleotidyltransferase, ZCCHC14, PAPD5/7, TENT4A/B, RG7834, antiviral therapy

## Abstract

Both hepatitis B virus (HBV), an hepadnavirus with a DNA genome, and hepatitis A virus (HAV), a picornavirus, require the TRAMP-like host ZCCHC14-TENT4 complex for efficient replication. However, whereas HBV requires the nucleotidyltransferase activity of TENT4 to extend and stabilize the 3′ poly(A) tails of mRNA transcribed from its genome, the role played by TENT4 in HAV replication is uncertain. HAV proteins are synthesized directly from its genomic RNA, which possesses a 3′ poly(A) tail, with its length and composition presumably maintained by 3D^pol^-catalyzed RNA transcription during its replicative cycle. Using nanopore long-read sequencing of RNA from infected cells, we confirm here that the length of the HAV 3′ poly(A) tail is not altered by treating infected cells with RG7834, a small molecule TENT4 inhibitor with potent anti-HAV activity. Despite this, TENT4 catalytic activity is essential for HAV replication. Surprisingly, nanopore sequencing revealed a low abundance of HAV subgenomic RNAs (hsRNAs) that extend from the 5′ end of the genome to a site within the 5′ untranslated RNA (5′UTR) immediately downstream of a stem-loop to which the ZCCHC14-TENT4 complex is recruited. These hsRNAs are polyadenylated, and their abundance is sharply reduced by RG7834 treatment, implying they are likely products of TENT4. Similar subgenomic RNAs were not identified in poliovirus-infected cells. hsRNAs are present not only in HAV-infected cell culture but also in the liver of HAV-infected mice, where they represent 1–3% of all HAV transcripts, suggesting their physiological relevance. However, transfecting exogenous hsRNA into TENT4-depleted cells failed to rescue HAV replication, leaving the functional role of hsRNA unresolved. These findings reveal a novel picornaviral subgenomic RNA species while highlighting mechanistic differences in the manner in which HAV and HBV exploit the host ZCCHC4-TENT4 complex for their replication.

## 1. Introduction

Hepatitis A virus (HAV) is a single-stranded positive-sense RNA virus classified within the genus *Hepatovirus* of the family *Picornaviridae* that is a common cause of acute, enterically transmitted viral hepatitis worldwide [1]. Like all picornaviruses, the RNA genome of HAV is single-stranded, positive-sense, and terminates in a 3′ poly(A) tail. Recent genome-wide CRISPR screens by us and others have identified numerous host factors essential for HAV replication, including, among them, the ZCCHC14-TENT4A/B complex [2,3,4]. ZCCHC14 is a Zn-finger RNA-binding protein, whereas TENT4A and TENT4B (also known as PAPD7 and PAPD5) are two paralogs of the TENT4 terminal nucleotidyltransferase that function as noncanonical poly(A) polymerases. Interestingly, ZCCHC14-TENT4 is also an essential host factor for hepatitis B virus (HBV) [5,6]. This is surprising, as HAV and HBV, an hepadnavirus with a DNA genome and a reverse transcription-based replication cycle, have no close phylogenetic relationship and differ dramatically in replication strategy.

The role of ZCCHC14-TENT4 in HBV infection is well understood [5,7,8]. ZCCHC14 binds to a stem-loop structure within the 3′ untranslated RNA (UTR) segment of HBV mRNAs to which it recruits TENT4. TENT4 subsequently catalyzes a “mixed-tailing” reaction that lengthens the 3′ poly(A) tail while introducing occasional guanosines with the overall effect of stabilizing the mRNA. This mRNA-stabilizing activity of the ZCCHC14-TENT4 complex is critical for the replication of HBV and inhibited by RG7834, a small molecule inhibitor of TENT that shortens the poly(A) tail and destabilizes HBV mRNAs [6,9]. Although HAV replication is similarly dependent upon the ZCCHC14-TENT 4 complex, the underlying mechanism appears to be quite different. ZCCHC14 binds to a small stem-loop (Vb) within the 5′UTR segment of the HAV genome that is structurally similar to the HBV stem-loop that binds the complex [4]. TENT4 is subsequently recruited to the 5′UTR by ZCCHC14. However, there is no evidence that TENT4 either extends the length of the HAV 3′ poly(A) tail or stabilizes the RNA. RG7834 does not reduce the length of the poly(A) tail in HAV-infected cells but acts instead to directly suppress viral RNA synthesis [4,10]. Exactly how TENT4 promotes viral RNA synthesis remains unknown.

Subgenomic viral RNAs (sgRNAs) are produced by members of several different families of positive-sense RNA viruses, including flaviviruses [11,12], alphaviruses [13], and coronaviruses [14]. These sgRNAs arise by mechanisms involving discontinuous transcription (coronaviruses), internal initiation (alphaviruses), and exonuclease degradation (flaviviruses). Subgenomic flaviviral RNAs (sfRNAs), in particular, play important roles in viral pathogenesis, including modulating host innate immune responses to infection and influencing the fate of infected cells [15,16,17]. Thus far, there have been no reports of subgenomic RNAs being generated by picornaviruses. Here, we show that HAV produces subgenomic RNAs (hepatovirus subgenomic RNA or hsRNA) from its 5′UTR. These approximately 700 nt long hsRNAs are 5′ co-terminal with the positive-sense viral genome and terminate at their 3′ ends with non-templated poly(A) tails. Their production is highly sensitive to TENT4 inhibition with RG7834 and decreases in hsRNA abundance correlate well with the antiviral effects of RG7834. We show that hsRNAs are present within the livers of HAV-infected mice, but whether they have a function in HAV replication or the modulation of host responses to HAV remains to be determined.

## 2. Materials and Methods

**Cells and viruses**. Human hepatoma-derived Huh-7.5 cells [18] were maintained in DMEM with 10% fetal bovine serum and 1% penicillin/streptomycin. 293T cells were obtained from ATCC. Cells tested negative for mycoplasma by a PCR assay (LookOut Mycoplasma PCR Detection Kit, Sigma-Aldrich). The cell-culture-adapted HM175 strain HAV variant 18f (GenBank KP879216.1) [19] and the 18f-NLuc reporter virus [20] were generated from infectious molecular cDNA clones and have been described previously. Murine infections were carried out with the mouse-passaged HM175 strain HAV [4,21]. Poliovirus infections were carried out with the type 1 Mahoney strain virus (GenBank V01149.1).

**siRNAs, plasmids, and antibodies**. siRNAs targeting TENT4A (5′-GGAGUGACGUUGAUUCAGA-3′) and TENT4B (5′-GGACGACACUUCAAUUAUU-3′) and non-targeting control siRNA were purchased from Horizon Discovery. shRNAs targeting TUT4 (TUT4#1, 5′-GAGGAAACGTGTCCGAGTA-3′; TUT4#2, 5′-ACAAGAATGCTAGCTACTT-3′) and TUT7 (TUT7#1, 5′-AGGATTTTCCAGGAACTAA-3′; TUT7#2, 5′-ACCATGAAAGTGTTCACAA-3′), and a non-targeting control (5′-ATCTCGCTTGGGCGAGAGTAAG-3′), were purchased from Horizon Discovery. Plasmids expressing wildtype TENT4A, TENT4B, or the catalytic mutants TENT4A-352A and TENT4B-326A were kindly provided by Dr. Narry Kim (Seoul National University, South Korea). The siRNA target sites in these plasmids were ablated by site-directed mutagenesis. TENT4A (HPA045487), TENT4B (HPA042968), and β-actin (A2228) antibodies were purchased from Millipore; TUT4 (18980-1-AP) and TUT7 (25196-1-AP) antibodies were purchased from Proteintech. IRDye 800CW- and IRDye 680RD-conjugated secondary antibodies were obtained from Li-Cor Biosciences.

**Luciferase assays.** Cells were lysed in 1× passive lysis buffer (Promega, #E1941) for 15 min at room temperature; then, lysates were transferred to opaque white 96-well plates (Corning, #3912). NLuc activity was quantified using the NLuc GLOW Assay kit (Nanolight Technology, #325), and luminescence was measured using a Biotek Synergy II multi-mode plate reader (BioTek Instruments).

**Transfections.** Synthetic RNAs were transcribed in vitro from linearized plasmid (for HAV genome RNA) or PCR-generated template DNA (for hsRNA) using the T7 RiboMAX™ Express Large Scale RNA Production System (Promega, #P1320). RNA transfections were carried out with TransIT-mRNA reagent (Mirus Bio, #MIR 2225) and plasmid transfections with TransIT-LT1 reagent (Mirus Bio, #MIR 2304) following the manufacturers’ suggested protocols.

**RT-qPCR**. Total RNAs were extracted from cultured cells using the RNeasy Kit (Qiagen) and from liver tissue using TRIzol reagent (Invitrogen Life Technologies) following the manufacturer’s protocol and used in cDNA synthesis reactions with the SuperScript III First-Strand Synthesis System (Invitrogen). Primers targeting the 5′ untranslated region of the HAV genome were used to quantify the HAV RNA genome (5′-ggtaggctacgggtgaaac-3′and 5′-aacaactcaccaatatccgc-3′) or hsRNA (5′-gattgactgtcggggct-3′and 5′-ttttttttttttttttttttaaggcc-3′), in an SYBR Green Real-Time qPCR assay (BioRad, #1725121) against a synthetic RNA standard. The primers 5′-gtcaccggagtccatcacg-3′ and 5′-gacccagatcatgtttgagacc-3′ were used to amplify β-actin mRNA as the control.

**Western blot**. Approximately 10^6^ 293T or Huh-7.5 cells were lysed in radioimmunoprecipitation assay (RIPA) buffer (Millipore, #20-188) for 20 min on ice and clarified by centrifugation at 14,000× *g* for 10 min at 4 °C. Lysate was mixed with 4× Laemmli buffer, incubated at 95 °C for 5 min and resolved in a 4–15% gradient SDS–polyacrylamide pre-cast gel (BioRad, #4561086). Proteins were transferred to a polyvinylidene fluoride membrane by semi-dry transfer using the Transblot Turbo apparatus (BioRad). Membranes were blocked in Odyssey Blocking Buffer (LI-COR Biosciences) and probed with a 1:1000 dilution of primary antibodies overnight. The membrane was washed with 0.05% Tween-20 in PBS and probed with a 1:10,000 dilution of goat anti-rabbit or anti-mouse secondary antibodies conjugated with IRDye 800 or IRDye 680 (LI-COR Biosciences) for 1 h at room temperature. Excess secondary antibodies were removed by washing with 0.05% Tween-20, and protein bands were visualized using an Odyssey Infrared Imaging System (LI-COR Biosciences).

**3′RACE assay.** A total of 10 µg of total RNAs extracted from infected cells were ligated to an RNA oligo linker (NEB, #S1315S) with T4 RNA ligase 2 (NEB, #M0242S). cDNAs were synthesized with the SuperScript III First-Strand Synthesis System (Invitrogen), with a primer containing the linker sequence 5′-ctacgtaacgattgatggtgcctacag-3′. PCR reactions were performed with a reverse primer for the linker sequence and specific forward primers to amplify the 3′ ends of HAV genomic RNA, 5′-agcatggcagagaagtaacg-3′, or hsRNA, 5′-ggtaggctacgggtgaaac-3′.

**Murine HAV infections.** *Ifnar1^−/−^* mice bred onto the C57BL/6 background were housed under specific pathogen-free conditions at the University of North Carolina at Chapel Hill in a facility managed by the Division of Comparative Medicine. Mice were kept in ventilated cages with standard food and water provided ad libitum and cared for in accordance with the policies and guidelines of the Institutional Animal Care and Use Committee (IACUC). HAV infection experiments followed an IACUC-approved protocol and were carried out as described previously [4].

**Poly(A) RNA purification.** Poly(A) tail-containing mRNA was purified from total RNA prior to making direct RNA sequencing libraries using Dynabeads™ Oligo(dT)_25_ beads (ThermoFisher, #61002). First, the beads were prepared by taking 20 µL of oligo(dT) beads per sample in a low-binding tube, and storage buffer was removed on a magnetic stand. The beads were washed with 40 µL of binding buffer (20 mM Tris-HCl pH 7.5, 1.0 M LiCl, and 2 mM EDTA) and resuspended in 40 µL of binding buffer. A total of 5 µg of total RNA from the control and treated samples was diluted with nuclease-free water up to volume of 40 µL and mixed with 40 µL of binding buffer. Total RNA was denatured by incubating at 65 °C for 2 min and mixed with 40 µL of pre-equilibrated oligo(dT) beads, followed by incubation at room temperature for 5 min. After incubation, beads were settled down on a magnetic stand for 5 min, and the supernatant was removed. Beads were washed twice with wash buffer (10 mM Tris-HCl, pH 7.5, 0.15 M LiCl, and 1 mM EDTA). After removing the wash buffer, the poly(A) tail-enriched mRNA was eluted in 10 µL of elution buffer (10 mM Tris-HCl, pH 7.5) by incubating the beads at 80 °C for 2 min and then incubating them on a magnetic stand.

**Direct RNA-sequencing library preparation.** Libraries were prepared using the standard protocol from Oxford Nanopore Technologies (ONT, #SQK-RNA002) with some minor modifications to incorporate barcode-tagged oligos. To detect the poly(A) tails and terminal residues of transcripts, a barcoded-oligonucleotide duplex mix (100 nM) was made, and a split-ligation strategy was used to ligate barcoded oligonucleotides to the 3′ end of RNA as described before [22,23]. For split ligation, ~8 µL of poly(A)-enriched RNA was mixed with 3 µL of ligation buffer (6% PEG8000 in 10X T4 DNA ligation buffer, NEB), 1.5 µL of T4 DNA ligase (NEB, # M0202L), 1.5 µL of barcoded oligo mix (100 nM), and 1 µL of 0.1 ng/µL RNA spike mix for library quality check [22,23] in a 15 µL total volume. The ligation reaction was incubated at room temperature for 60 min. To maximize sequencing throughput, the barcoded RNA was reverse-transcribed into an RNA/cDNA hybrid by mixing 15 µL of barcoded RNA with 9 µL of nuclease-free water, 2 µL of dNTPs (10 mM), 8 µL of 5X first-strand buffer, 4 µL of 100 mM DTT, and 2 µL of SuperScript II reverse transcriptase (ThermoFisher, #18064022). The reaction mix was incubated at 50 °C for 50 min and then 70 °C for 10 min, before being cooled to 4 °C in a thermocycler. The RNA/cDNA hybrid was purified using RNAClean XP beads (Beckman Coulter, #A63987) by mixing 72 µL of beads to each reaction and incubating at room temperature for 5 min. The supernatant was removed on a magnetic stand and the beads were washed with 150 µL of freshly prepared 70% ethanol. The beads were resuspended in 20 µL of nuclease-free water and incubated at room temperature for 5 min followed by elution on a magnetic stand.

The purified RNA/cDNA hybrid was then ligated to a motor protein (RMX, supplied with the kit ONT, #SQK-RNA002)-containing sequencing adapter. For this, 8 µL of ligation buffer (6% PEG in 10× T4 DNA ligation buffer, NEB), 6 µL of RMX-RNA adapter, 3 µL of nuclease-free water, and 3 µL of T4 DNA ligase (NEB, # M0202L) were mixed with 20 µL of purified RNA/cDNA hybrid in a final reaction volume of 40 µL. The ligation reaction was incubated at room temperature for 15 min.

The adapter-ligated RNA/cDNA hybrids were purified by mixing with 40 µL of RNAClean XP beads (Beckman Coulter, #A63987) and incubated at room temperature for 5 min. The beads were washed twice with 150 µL of wash buffer (WSB from ONT, #SQK-RNA002), and the washed beads were resuspended in 21 µL of elution buffer (EB from ONT, #SQK-RNA002). The beads were incubated at room temperature for 15 min, and the final libraries were eluted on a magnetic stand.

The libraries were prepared for loading into the ONT flow cell (R9.4.1) using the flow cell priming kit (ONT, #EXP-FLP002) according to the manufacturer’s instructions. Briefly, 21 µL of RNA libraries was mixed with 37.5 µL of RNA running buffer (RRB from ONT, #SQK-RNA002) and 17.5 µL of nuclease-free water in a 75 µL total volume. The flow cell was primed with 800 µL of the FLT/FB mix (30 µL FLT into 1 mL FB tube, from ONT, #EXP-FLP002) and incubated for 5 min, followed by an additional 200 µL of the FLT-FB mix through the priming port of the flow cell. After priming, 75 µL of direct RNA sequencing library was loaded through the spot-on port, and both ports were closed. Libraries were sequenced on an ONT MinION instrument, and Fast5 files were saved by ONT MinKNOW software, version 5.2.15.

**Nanopore sequence data analysis.** The direct RNA-seq library data generated on MinION instruments were base-called by Guppy (ONT). The reads were then aligned to the human transcriptome and HAV genome using minimap2 [24] and indexed with SAMtools [25]. The length of the poly(A) tail of each read was determined using Nanopolish [26,27]. Reads were filtered, and only those reads that had a poly(A) tail quality score (qc_tag = “PASS”) and a confidence interval (C.I.) greater than 0.95 were selected for further analysis. Reads were demultiplexed using DeePlexiCon [28], and the identity of terminal modifications were assigned according to the ligated barcodes (BC1 for unmodified tails, BC2 for monouridylation, BC3 for oligouridylation, and BC4 for guanylation).

## 3. Results

**Figure 1:**
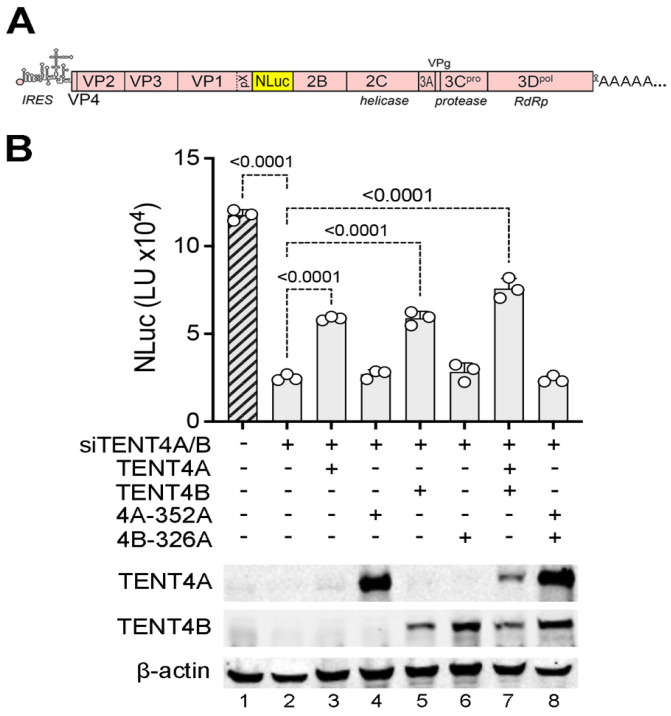
Reconstituting TENT4 expression in TENT4-depleted 293T cells partially rescues HAV replication. (**A**) Genome organization of the 18f-NLuc reporter virus [20]. (**B**) 293T cells were depleted of endogenous TENT4A/B by siRNAs, and recombinant TENT4A, TENT4B, or catalytic mutants TENT4A-352A and TENT4B-326A were expressed from vectors containing synonymous nucleotide changes conferring siRNA resistance. Cells were infected with 18f-NLuc virus for 48 h. Corresponding immunoblots are shown in the bottom. *p*-values determined by one-way ANOVA with Dunnett correction for multiple comparisons.

**TENT4 catalytic activity is required for HAV replication.** Although RG7834 has potent antiviral activity against HAV, it is not clear whether this results from its inhibition of TENT4 catalytic activity or disruption of the ZCCHC14-TENT4 complex [4]. To confirm a previous report suggesting that the catalytic activity of TENT4A/B is required for HAV replication [3], we reconstituted TENT4A or TENT4B expression with wildtype or catalytically inactive mutants in 293T cells that had been depleted of endogenous TENT4A/B by transfection of siRNA and assessed the capacity of these cells to support HAV replication using a recombinant virus that expresses a nanoluciferase reporter (18f-NLuc virus) [20] (Figure 1A). Consistent with previous results [4], RNAi-mediated depletion of endogenous TENT4A/B reduced HAV replication by ~5-fold (Figure 1B, lane 2). Reconstituting expression with either wildtype siRNA-resistant TENT4A or TENT4B proteins partially rescued HAV replication, whereas reconstituting expression with catalytic mutants of TENT4A (D352A), TENT4B (D326A), or both mutants was without effect (Figure 1B). These results confirm the need for TENT4 catalytic activity in HAV replication and show that this can be provided by either TENT4 isoform.

**Figure 2:**
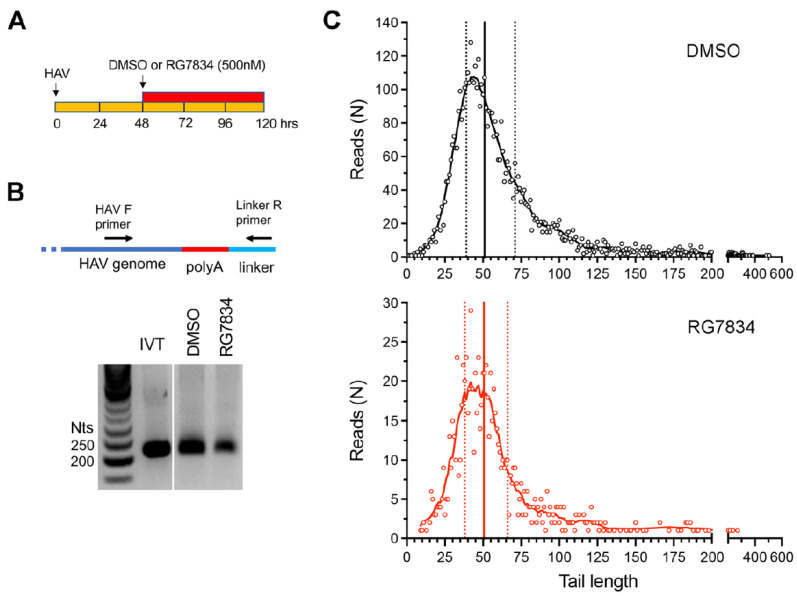
HAV 3′ poly(A) tail length distribution in infected Huh-7.5 cells. (**A**) Experimental scheme showing RG7834 (500 nM) or vehicle-only (DMSO) treatment of cells between 48 and 120 h after infection. (**B**) 3′RACE assay for HAV genomic RNA. IVT, in vitro transcribed RNA. (**C**) 3′ poly(A) tail lengths determined by nanopore sequencing. Data shown represent combined results from two independent experiments. The total numbers of HAV reads were 5106 for control cells and 909 for RG7834-treated cells. Vertical lines indicate quartiles: DMSO control cell median = 51 nts, RG7834-treated cells = 52 nts.

**HAV 3′ poly(A) tail composition and length in RG7834-treated cells.** Despite the need for TENT4 enzymatic activity in HAV replication, we and others have found no evidence that TENT4 regulates the length the HAV 3′ poly(A) tail [3,4]. This is consistent with the fact that the 3′ poly(A) sequence of HAV is intrinsic to its genome and is thus generated during positive-strand RNA synthesis by the viral 3D^pol^ RNA-dependent RNA polymerase. However, this is in sharp contrast to HBV, where the depletion of either ZCCHC14 or TENT4 shortens the length of the poly(A) tails of viral mRNAs [5]. To formally rule out a role for TENT4 in polyadenylating HAV genomic RNA, we carried out two additional assays. First, we used a 3′ RACE assay to assess HAV poly(A) tail length with or without treatment of infected Huh-7.5 cells with RG7834 (Figure 2A). RG7834 has an antiviral IC_50_ of 6–12 nM in these cells [4,29]. To assess 3′ tail length, an RNA linker was ligated to the 3′ end of the RNA, and the poly(A) tail of HAV RNAs subsequently amplified using an HAV-specific forward primer and a reverse primer complementary to the linker sequence (Figure 2B). An in vitro transcribed HAV RNA with a 40-nucleotide (nt) 3′ poly(A) tail served as control. These experiments demonstrated that, while RG7834 significantly reduces the abundance of HAV RNA, it does not alter the length of the poly(A) tail (Figure 2B).

**Figure 3:**
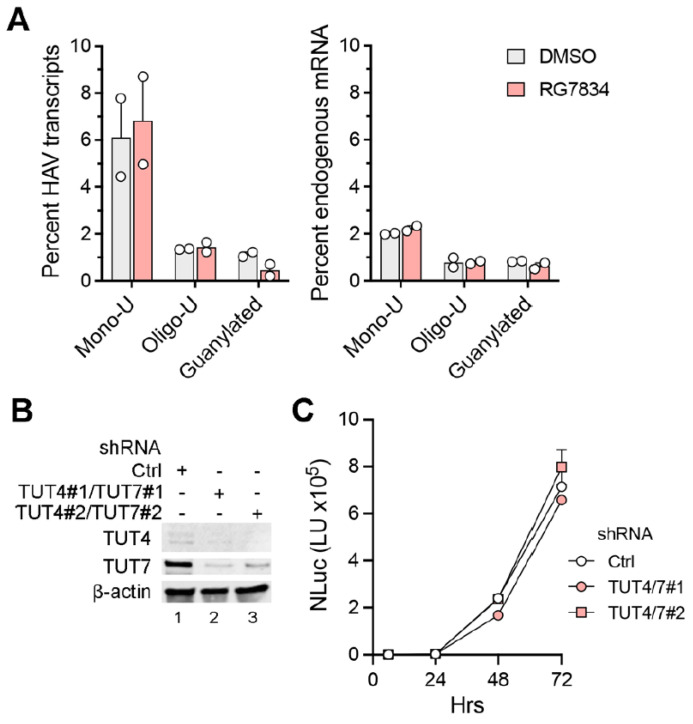
3′ poly(A) tail modifications in HAV RNA and host mRNA extracted from infected cells. (**A**) Percentage of mono-uridylated, oligo-uridylated, and guanylated HAV (**left**) or cellular RNAs (**right**) in the nanopore experiments. (**B**) Immunoblots showing shRNA knockdowns of TUT4 and TUT7 in Huh-7.5 cells. (**C**) Replication of 18f-NLuc virus in TUT4/7 knockdown cells. Error bars denote standard deviation of 3 technical replicates.

Additionally, we carried out nanopore long-read sequencing of RNAs from HAV-infected Huh-7.5 cells. This allowed us to capture the length of the poly(A) tail in each individual viral RNA. These results revealed a wide range of poly(A) tail lengths in HAV RNAs (Figure 2C, top), with the most abundant, modal length being ~42 nts and the median length being 51 nts. Despite a sharp decline in HAV RNA abundance, there was no change in the distribution of poly(A) tail lengths in RG7834-treated cells (Figure 2C, bottom). These results thus confirm that TENT4 does not regulate the length of the HAV 3′ poly(A) tail. Interestingly, the nanopore sequencing data revealed a small fraction (6–7%) of viral RNAs possess 3′ tails that are terminally mono- or oligo-uridylated (Figure 3A, left). Such 3′ uridylation is a signature of the cellular terminal uridyltransferases TUT4 and TUT7, which are not inhibited by RG7834, and it generally marks the RNA for degradation [30]. Interestingly, the proportion of HAV RNAs that were uridylated was substantially greater than the proportion of endogenous host cell mRNAs in these experiments (*p* < 0.05 by two-way ANOVA) (Figure 3A, right). Uridylation of poly(A) tails typically promotes RNA degradation, but shRNA knockdown of TUT4 and TUT7 had no apparent effect on 18f-NLuc virus replication in Huh-7.5 cells (Figure 3B,C). A smaller fraction of the viral RNAs (about 1%) were guanylated at the 3′ terminal or penultimate base, a signature of TENT4 known as “mixed tailing”, which acts to shield the RNA from deadenylation and degradation [31]. The percentage of guanylated viral tails was reduced in RG7834-treated cells, but this difference did not reach statistical significance.

**Figure 4:**
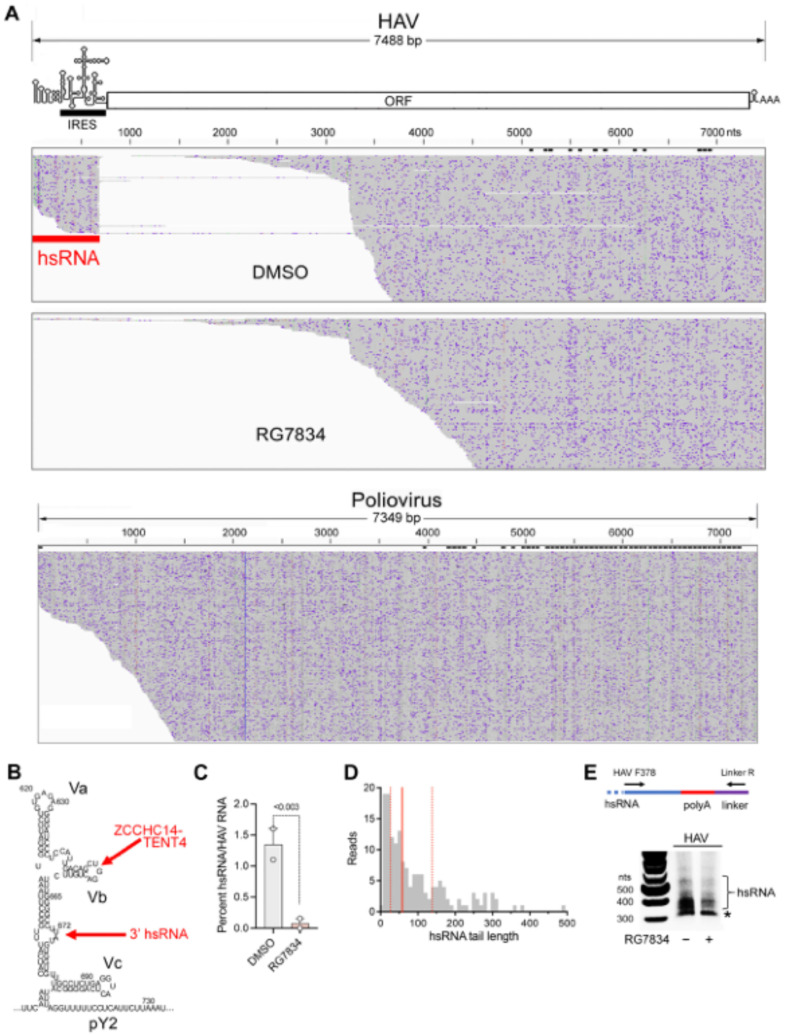
Subgenomic hsRNAs are expressed in HAV-infected Huh-7.5 cells. (**A**) (**top**) Alignment plot of long-read nanopore sequences of HAV RNA isolated from RG7834 and DMSO-treated Huh-7.5 cells. 3′ poly-adenylated subgenomic hsRNAs (red band) align with the 5′ untranslated RNA (5′UTR) segment of the genome in cells treated with the DMSO vehicle. hsRNAs are not detected among RNAs isolated from cells treated with the TENT4 inhibitor RG7834. hsRNAs are 5′ co-terminal with the genome and overlap the internal ribosome entry site (IRES, black band). (**bottom**) Similar nanopore sequencing of poliovirus-infected cells. (**B**) Predicted secondary RNA structure of domain V within the 5′ UTR [32], showing stem-loops Va, Vb (to which ZCCHC14 is recruited) [4], and Vc, and the position at which hsRNAs terminate, nts 672–3 (numbering is for wildtype HM175 virus, GenBank M14707.1). pY2, second pyrimidine-rich track. (**C**) hsRNA abundance relative to HAV RNA transcripts in two independent experiments. HAV reads in DMSO control cells ranged from 3266 to 3701, and those in RG7834-treated cells ranged from 611 to 639. *p*-values < 0.003 by X^2^ contingency test in both experiments. (**D**) hsRNA 3′ poly(A) tail length. Vertical lines represent quartiles; median = 58 nts. (**E**) 3′RACE assay for hsRNA. Treatment scheme is the same as in Figure 2A. * Nonspecific mitochondrial RNA.

**Subgenomic HAV RNAs are generated in infected cells.** A striking observation emerging from the nanopore sequencing experiments was the existence of a population of short viral transcripts sharing their 5′ end with the positive-strand RNA genome and aligning with the 5′UTR (Figure 4A, top). These short transcripts, which we refer to as hepatovirus subgenomic RNA (hsRNA), are 3′ polyadenylated, with the HAV sequence terminating at nucleotide 672 (U) or 673 (A) (nucleotides 684 and 685 in the cell-culture-adapted 18f and 18f-NLuc reporter viruses). This position is 11–12 bases downstream of stem-loop Vb (SL-Vb), to which ZCCHC14 binds [4], and within a small bulge-loop that is separated from SL-Vb by a highly stable duplex [32] (Figure 4B). Terminating at this location within the 5′UTR, these hsRNAs thus appear to present a perfect substrate for polyadenylation by the ZCCHC14-TENT4 complex. The hsRNAs represented about 1.5 percent of all HAV reads identified in infected cells. Importantly, the proportion of hsRNAs was reduced to <0.2% by RG7834 treatment (Figure 4C), strongly suggesting they are stabilized by TENT4 polyadenylation. Equally importantly, similar nanopore sequencing of poliovirus-infected Huh-7.5 cells failed to demonstrate similar subgenomic poliovirus transcripts (Figure 4A, bottom), suggesting hsRNAs are generated by an HAV-specific process. Targeted analysis of the hsRNA reads revealed they contain variable length 3′ poly(A) tails, with a median length of 58 nts and no clear modal length (Figure 4D). Similar to HAV RNA (Figure 3A), 8.9% of the hsRNA transcripts were 3′ mono-uridylated and 1.9% were oligo-uridylated; none were guanylated. We developed a 3′ RACE assay to analyze the poly(A) tails of hsRNA similar to the assay shown in Figure 2B for genomic RNA (Figure 4E). This assay confirmed a median hsRNA tail length of approximately 60 nts. The abundance of hsRNAs was reduced by RG7834 treatment without an obvious accumulation of hsRNAs with shorter-length poly(A) tails (Figure 4E).

**Figure 5:**
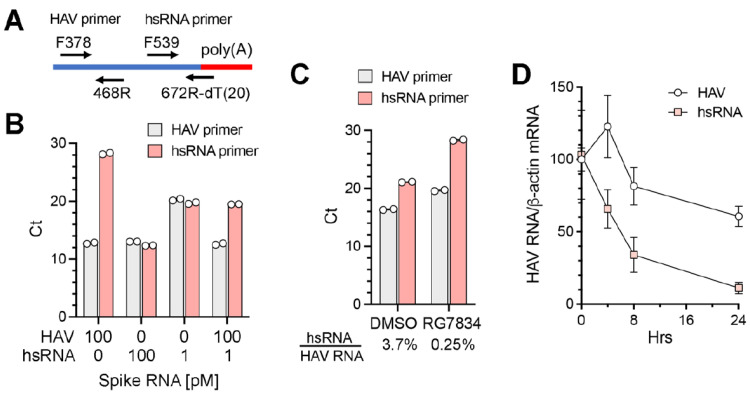
Quantitative RT-PCR assay for hsRNA. (**A**) Diagram demonstrating the qPCR primer sets for HAV genomic RNA and hsRNA. The reverse primer for hsRNA contains 20nt oligo-dT followed by 6nts complementary to nts 667–672 of the wildtype HAV genome. (**B**) Threshold cycles (Ct values) from qPCR assays spiked with synthetic HAV or hsRNA RNA containing 30 nt poly(A) using the HAV or hsRNA primer sets. (**C**) Ct values from qPCR assays of RNAs samples extracted from HAV-infected Huh-7.5 cells. RG7834 treatment was as in Figure 2A. (**D**) Huh-7.5 cells were infected with 18f virus for 2 days and treated with 500 nM RG7834. HAV and hsRNA levels at indicated time points were determined by qRT-PCR and normalized to β-actin mRNA. The RNA levels at time 0 h were arbitrarily set to 100. Error bars denote standard deviation (n = 3).

To better quantify the abundance of hsRNAs in infected cells under various conditions, we developed a qPCR assay specific for hsRNAs (Figure 5A). The reverse primer for hsRNAs comprised a 20 nt dT oligomer followed by 6 nts complementary to nt667–672 of the HAV genome (nt679–684 of the 18f virus). When tested with synthetic RNAs, the hsRNA primer set specifically amplified hsRNA with only minimal amplification of full-length positive-strand genomic RNA; in contrast, the HAV primer set amplified both RNAs equally (Figure 5B). A quantitative difference was evident when the synthetic hsRNA concentration was reduced from 100 pM to 1 pM in the reaction. Importantly, when the hsRNA and genomic RNA were mixed at a ratio of 1:100, which approximates the composition in infected cells, the hsRNA primer set accurately measured the abundance of hsRNA with minimal interference from the genomic RNA (Figure 5B). We used this assay to characterize RNA extracted from HAV-infected Huh-7.5 cells. The hsRNA/genomic RNA ratio measured by qPCR was 3.7% in infected cells (Figure 5C). RG7834 treatment reduced the genomic RNA abundance by ~8-fold while reducing the hsRNA abundance > 100-fold, resulting in a hsRNA/genomic RNA ratio of 0.25% (Figure 5C). This result is in line with the nanopore sequencing results shown in Figure 4C. Since TENT4 polyadenylation is associated with enhanced stability for both cellular and viral RNAs [8,31], we speculate that the greater reduction in hsRNA abundance following RG7834 treatment reflects enhanced decay due to reduced TENT4 polyadenylation. To explore this hypothesis, we assessed the decay of hsRNA after RG7834 treatment over a time course of 24 h. As shown in Figure 5D, the hsRNA degrades more rapidly (half-life ~ 6 h) than HAV genome RNA (half-life > 24 h) following the initiation of RG7834 treatment. These results are consistent with the notion that TENT4 polyadenylates and stabilizes hsRNAs.

**Figure 6:**
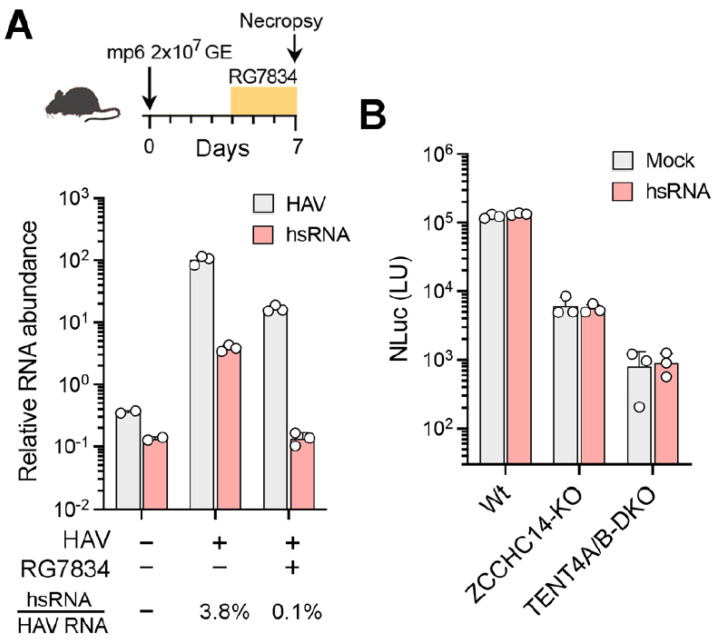
hsRNAs are present in vivo in infected mice but do not rescue viral replication in ZCCHC14-TENT4-depleted hepatoma cells. (**A**) (**top**) Outline of HAV infection in *Ifnar1^−/−^* mice inoculated intravenously with mouse-passaged mp6 virus and treated with RG7834 or DMSO vehicle only, administered by oral gavage at 10 µg/kg twice daily between days 4 and 7 post-infection. (**bottom**) qRT-PCR quantitation of genomic HAV RNA and hsRNA in infected mouse livers 7 days post-infection, with or without RG7834 treatment. RNA levels were normalized to β-actin mRNA. HAV RNA levels in infected mice without treatment were arbitrarily set to 100 (n = 3). (**B**) Wildtype Huh-7.5, ZCCHC14-KO, and TENT4A/B-DKO cells were transfected with synthetic hsRNA for 2 h and infected with the 18f-NLuc reporter virus. NLuc activities were measured at 72 h post-infection (n = 3).

**hsRNAs are present within the liver of HAV-infected mice.** HAV readily infects type I interferon receptor knockout (*Ifnar1^−/−^*) mice, resulting in elevated serum alanine aminotransferase (ALT) activity, hepatic inflammation, and fecal shedding of virus, effectively recapitulating human hepatitis A [21]. To determine whether hsRNAs are produced by HAV in vivo, we extracted RNA from the liver of HAV-infected *Ifnar1^−/−^* mice 7 days after infection and assessed the presence of hsRNAs using the hsRNA-specific qRT-PCR assay described above. hsRNAs were readily detected in the infected liver tissue, constituting approximately 3.8% of all HAV transcripts (Figure 6A). In mice treated for 48 h with RG7834 beginning on day 4 following infection, the abundance of HAV RNA was reduced by ~10-fold while hsRNAs were reduced more than 30-fold and were no longer detectable (Figure 6A). These results indicate that hsRNAs are present in vivo within infected livers and are quantitatively reduced by treatment with RG7834.

Taken collectively, the data described above show that hsRNAs are polyadenylated by TENT4 in HAV-infected cells and that RG7834 inhibition of TENT4 has a more profound effect on hsRNA abundance than the abundance of viral genomic RNA. This raises two critical questions: do hsRNAs play an essential role in the incompletely understood HAV replicative cycle, and is the antiviral efficacy of RG7834 due to its capacity to destabilize and reduce hsRNA abundance? Or, alternatively, are hsRNAs of no consequence in the replicative cycle and simply reflective of ZCCHC14-TENT4 recruitment to the 5′UTR for some other purpose, perhaps to promote genome circularization via interactions with the viral 3′UTR as we have suggested previously [4]? In an effort to gain insight into this question, we asked whether providing exogenous hsRNA would enhance viral replication or rescue replication in TENT4-depleted cells. Accordingly, in vitro transcribed hsRNAs with a 30 nt 3′ poly(A) tail were transfected into wildtype, ZCCHC14-depleted (ZCCHC14-KO), or TENT4A/B-depleted (TENT4A/B-KO) Huh-7.5 cells [4], and this was followed by infection with the 18f-NLuc reporter virus for 72 h. The transfected hsRNAs had no apparent effect on NLuc activity in wildtype cells, nor did they rescue viral replication in either knockout cell line (Figure 6B). Despite these negative data, a role for hsRNAs in HAV replication cannot be entirely ruled out (see Section 4 Discussion).

## 4. Discussion

The noncanonical TENT4 poly(A) polymerases, TENT4A (known variously as PAPD7, LAK1, TRF4-1, or TUT5) and TENT4B (PAPD5, TRF4-2, or TUT3), form complexes with different RNA-binding partners, such as ZCCHC7, ZCCHC14, and ZCCHC2, to target specific RNAs [8,33,34,35]. The ZCCHC14-TENT4 complex is particularly interesting for its function in virus infections. ZCCHC14-TENT4 polyadenylates HBV and human cytomegalovirus (HCMV) RNA transcripts, incorporating occasional guanosines that enhance transcript stability by suppressing 3′ exosome-mediated decay [5,8]. Chemical inhibition of this activity has potent antiviral effects against HBV [36]. Despite being recruited to the HAV genome and despite its essential role in HAV replication, previous studies suggest the ZCCHC14-TENT4 complex does not act similarly by stabilizing HAV RNA [3,4]. In this study, we confirmed that chemical inhibition of TENT4 does not alter the length or composition of the HAV 3′ poly(A) tail using two orthogonal approaches: a 3′RACE assay and nanopore long-read sequencing (Figure 2).

Nanopore sequencing revealed previously unknown aspects of the HAV poly(A) tail. The modal length of the 3′ tail length was ~42 nts, and its median length was 51 nts, with a small percentage of tails exceeding 400 nts in length. Many of the RNA transcripts have 3′ tails that are lengthier than the 3′ poly(A) tail present in RNA transcribed in vitro from the infectious molecular clone from which the 18f virus was generated (40 nts). This indicates the existence of a mechanism by which HAV poly(A) tails can be extended in infected cells. However, this mechanism does not appear to be dependent upon TENT4 activity as RG7834 treatment did not alter the distribution of tail lengths (Figure 2C). We speculate that extension of the poly(A) tail is catalyzed by the viral polymerase, 3D^pol^, either in a non-templated manner or by the polymerase sliding back on the negative-strand template. Similar to endogenous mRNA, the nanopore sequencing also revealed 3′ terminal modifications of the HAV poly(A) tail (Figure 3A). Interestingly, HAV RNAs were uridylated significantly more frequently than endogenous RNAs. It is possible that this higher frequency of uridylation is due to the exclusive cytoplasmic localization of HAV RNA, as the uridyltransferases that are likely responsible for this, TUT4 and TUT7, are primarily cytoplasmic. Although uridylation generally marks cellular mRNAs for degradation [30], uridylation of the HAV poly(A) tail appears to be inconsequential for HAV infection, as knocking down TUT4/7 expression had no effect on viral replication (Figure 3C).

The discovery of subgenomic hsRNAs aligning with the 5′ end of the HAV genome in infected cells was both surprising and intriguing (Figure 4). Although subgenomic RNAs are generated by other RNA viruses and play an important role particularly in flavivirus pathogenesis [15,16], subgenomic RNAs are not known to be produced by any picornavirus. We confirmed the absence of similar subgenomic transcripts in poliovirus-infected cells (Figure 4A). Thus, the production of a defined subgenomic RNA species appears to be specific to hepatoviruses, as is the HAV requirement for ZCCHC14-TENT4 for viral RNA synthesis [4]. hsRNAs are 5′ co-terminal with HAV genomic RNA. They are 3′ polyadenylated, with their 3′ terminus aligning with a bulge-loop in the 5′UTR a short distance downstream of stem-loop SL-Vb, to which ZCCHC14 binds and recruits TENT4 (Figure 4B). We speculate that the 3′-5′ processing of the 3D^pol^ polymerase along the negative-strand RNA template during positive-strand synthesis is sufficiently slow and perhaps paused by RNA structure downstream of stem-loop Vb, to allow the stem-loop to assume its fold and bind the ZCCHC14-TENT4 complex. TENT4 then snatches the nascent positive-strand RNA from the HAV polymerase and polyadenylates it. Consistent with this hypothesis, both the ratio of hsRNA to genomic RNA and the length of the 3′ poly(A) tails of hsRNA are reduced by RG7834 treatment of infected cells (Figure 4D,E).

An alternative—but, as we believe, less likely—possibility is that hsRNAs are generated from lengthier (perhaps full-length) viral RNAs by exonuclease digestion in a process similar to the production of subgenomic flavivirus RNA (sfRNA). sfRNAs are generated from full-length flavivirus transcripts by the 5′-3′ exonuclease Xrn1, which progressively degrades the RNA until stalled by RNA structure just upstream of the 3′UTR [37,38]. In the case of HAV, the full-length viral RNA could be digested by a 3′-5′ exonuclease that is stalled by the ZCCHC14-TENT4 complex bound to SL-Vb and then polyadenylated by TENT4. Both models are plausible and worthy of further investigation. Endonucleolytic cleavage of full-length genomic RNA, followed by polyadenylation of the 5′ cleavage fragment, seems much less plausible.

hsRNAs are present both in cell culture and in the liver of HAV-infected mice (Figure 6A), suggesting they may be physiologically relevant. Since the production of hsRNA is unique to HAV, and RG7834 is not active against other members of the picornavirus family, it is tempting to link the antiviral action of RG7834 to its inhibition of TENT4 production and stabilization of hsRNA. However, it is not clear how hsRNAs might contribute to the viral life cycle. We do not know if they are linked to VPg at their 5′ end or whether they are packaged in capsids and exported from cells. There are several short ORFs within hsRNA that could encode polypeptides between 10 and 30 amino acids in length. All of these ORFs begin with a noncanonical initiation codon (UUG or GUG), and it is uncertain how any could be translated in the absence of a 5′ m7G cap or a functionally intact internal ribosome entry site (IRES). Since the 5′ and 3′ boundaries of the HAV IRES map between nts 150 and 248 and between nts 695 and 732 (Figure 4A) [39], hsRNAs (nts 1–672) contain only part of the IRES.

hsRNAs constitute only a small percentage (1–3%) of viral transcripts but, nonetheless, are much more abundant than the negative-strand RNA intermediate (~0.1% of viral transcripts) that templates positive-strand synthesis [40]. Could they somehow positively regulate viral RNA synthesis, or does the ZCCHC14-TENT4 complex have an additional, essential role in replication beyond polyadenylating and stabilizing hsRNA? Transfecting in vitro transcribed hsRNA into TENT4 or ZCCHC14 knockout cells failed to rescue viral replication (Figure 6B). However, this does not rule out an important role for hsRNA in RNA replication, since the membranous replication organelle within which RNA synthesis occurs is likely to be inaccessible to exogenous transfected RNA. Or hsRNAs might act in cis during viral RNA synthesis and, thus, cannot be *trans*-complemented. Alternatively, is it possible that hsRNAs contribute to viral infection by antagonizing intrinsic antiviral defense mechanisms, similar to how sfRNA generated by dengue virus interacts with the ubiquitin ligase TRIM25 to block interferon production [41]? Clearly, much more work will need to be conducted to elucidate the role of hsRNAs in HAV infection.

## Data Availability

Nanopore sequencing data have been deposited in the GEO database with the accession number GSE293395. All other data are available in the manuscript and figures.
